# The Potential of Targeting Autophagy-Related Non-coding RNAs in the Treatment of Alzheimer’s and Parkinson’s Diseases

**DOI:** 10.1007/s10571-024-01461-w

**Published:** 2024-03-10

**Authors:** Abdolkarim Talebi Taheri, Zakieh Golshadi, Hamidreza Zare, Azam Alinaghipour, Zahra Faghihi, Ehsan Dadgostar, Zeinab Tamtaji, Michael Aschner, Hamed Mirzaei, Omid Reza Tamtaji, Fatemeh Nabavizadeh

**Affiliations:** 1 Students’ Scientific Research Center, Tehran University of Medical Sciences, Tehran, Iran; 2 Department of Clinical Biochemistry, School of Medicine, Tehran University of Medical Sciences, Tehran, Iran; 3 Student Research Committee, Qazvin University of Medical Sciences, Qazvin, Iran; 4 University of Szeged, Szeged, Hungary; 5 School of Medical Sciences, Yazd Branch, Islamic Azad University, Yazd, Iran; 6 Department of Physiology, School of Medicine, Tehran University of Medical Sciences, Tehran, I.R. of Iran; 7 Behavioral Sciences Research Center, Isfahan University of Medical Sciences, Isfahan, I.R. of Iran; 8 Student Research Committee, Isfahan University of Medical Sciences, Isfahan, I.R. of Iran; 9 Student Research Committee, Kashan University of Medical Sciences, Kashan, I.R. of Iran; 10 Department of Molecular Pharmacology, Albert Einstein College of Medicine, Bronx, NY 10461 USA; 11 Research Center for Biochemistry and Nutrition in Metabolic Diseases, Kashan University of Medical Sciences, Kashan, I.R. of Iran; 12 Electrophysiology Research Center, Neuroscience Institute, Tehran University of Medical Sciences, Tehran, I.R. of Iran

**Keywords:** Neurodegenerative disorders, Non-coding RNAs, Autophagy, microRNA, Long non-coding (lnc) RNA

## Abstract

Clearance of accumulated protein aggregates is one of the functions of autophagy. Recently, a clearer understanding of non-coding RNAs (ncRNAs) functions documented that ncRNAs have important roles in several biological processes associated with the development and progression of neurodegenerative disorders. Subtypes of ncRNA, including microRNA (miRNA), long noncoding RNA (lncRNA), and circular RNA (circRNA), are commonly dysregulated in neurodegenerative disorders such as Alzheimer and Parkinson diseases. Dysregulation of these non-coding RNAs has been associated with inhibition or stimulation of autophagy. Decreased miR-124 led to decreased/increased autophagy in experimental model of Alzheimer and Parkinson diseases. Increased BACE1-AS showed enhanced autophagy in Alzheimer disease by targeting miR-214-3p, Beclin-1, LC3-I/LC3-II, p62, and ATG5. A significant increase in NEAT1led to stimulated autophagy in experimental model of PD by targeting PINK1, LC3-I, LC3-II, p62 and miR-374c-5p. In addition, increased BDNF-AS and SNHG1 decreased autophagy in MPTP-induced PD by targeting miR-125b-5p and miR-221/222, respectively. The upregulation of circNF1-419 and circSAMD4A resulted in an increased autophagy by regulating Dynamin-1 and miR-29c 3p, respectively. A detailed discussion of miRNAs, circRNAs, and lncRNAs in relation to their autophagy-related signaling pathways is presented in this study.

**Graphical Abstract:**

Autophagy-related non-coding RNAs in neurodegenerative diseases.

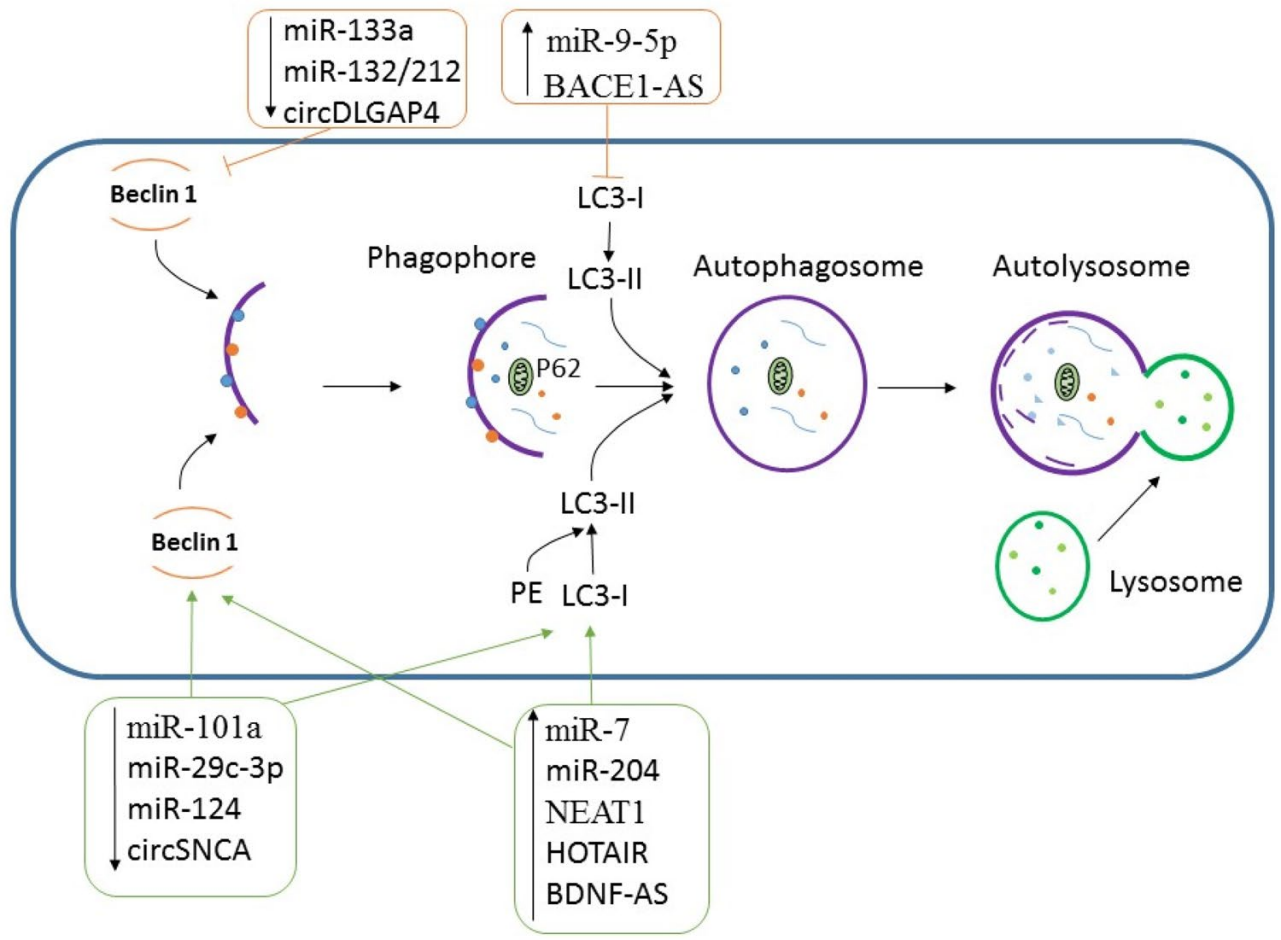

## Introduction

The term “neurodegenerative disease (ND)” describes the progressive loss of specific populations of neurons by toxic or metabolic processes. There are several means to classify neurodegenerative diseases by their primary clinical features, anatomic distributions of neurodegeneration, or molecular abnormalities (Dugger and Dickson 2017). Alzheimer’s disease (AD) and Parkinson’s disease (PD) are two major NDs (Checkoway et al. 2011). As the ability of the central nervous system (CNS) for regeneration is limited, it is essential to limit injury within this organ (Kuhn et al. 2001).

A cell’s autophagy process involves the transport of cytosolic components, macromolecules, viruses, bacteria, and organelles to lysosomes for destruction (Patergnani and Pinton 2015). Physiological and pathological conditions are impacted by autophagy and its specialized forms. The function of autophagy in normal conditions is to remove unnecessary material, regulate organelle turnover, and meet energy demands. Autophagy can have both beneficial and harmful effects on pathological conditions (Patergnani et al. 2020; Xue et al. 2020). The clearance of accumulated protein aggregates is one of the functions of autophagy that contributes to neuronal function and neurodegenerative disorders (Nah et al. 2015). A wide range of neurodegenerative diseases is characterized by the accumulation of proteins (Menzies et al. 2015; Ravikumar et al. 2002), with autophagy contributing to disease progression (Menzies et al. 2015) and neuronal loss (Hara et al. 2006; Komatsu et al. 2006; Levine and Kroemer 2008; Nikoletopoulou et al. 2013).

Studies have focused on evaluating specific biomarkers related to genes that encode autophagy pathways-related proteins in neurodegenerative diseases. In this regard, through their ability to adjust the translation of other RNAs, non-coding RNAs (ncRNAs) contribute to the generation of functional proteins through the effect on the expression of protein-coding transcripts (Esteller 2011). A series of cleavage events produce mature microRNAs after RNA polymerases II and III transcribe microRNA precursors. lncRNAs also contain a poly (A) terminus at 3′ and a 5′ methyl-cytosine cap which is transcribed by RNA polymerase II (Pol II). lncRNAs biogenesis is similar to that of mRNA with a few variations in the process. RNA circular molecules (circRNAs) are endogenous non-coding RNAs generated by the back-splicing process. Some circRNAs consisting of introns originate in the nucleus, while others have one or more exons with major locations in the cytoplasm. A circular RNA does not have a polyadenylated tail or a 5–3′ direction. These characteristics render ncRNAs more stable in plasma and tissues as they possess a continuous loop of covalent bonds. ncRNAs can be useful in diagnostic and prognostic goals and have roles in regulating autophagy pathways in neurodegenerative diseases (Shah et al. 2018; Xu et al. 2020).

Herein, we highlight the role of several ncRNAs associated with autophagy in AD and PD as neurodegenerative diseases. miRNAs, circRNAs, and lncRNAs will be discussed in detail. Due to the importance of RNAs for neurodegenerative diseases, multiple studies have been conducted on ncRNAs; however, in the present review, the effects of these ncRNAs on autophagy-related genes and pathways in AD and PD are discussed, distinguishing this review from others.

## Autophagy and Neurodegenerative Diseases

The biochemist Christian de Duve introduced the term autophagy in late 1963, referring to cellular degradation and recycling via a self-degradative pathway (Duve and Wattiaux, 1966). In mammalian cells, macroautophagy is the best-characterized and most prevalent mode of autophagy. Macroautophagy occurs when the expanding phagophore sequesters random cytoplasm and dysfunctional organelles, leading to autophagosome formation. Following autophagosome fusion with the vacuole membrane, the autophagic body enters the vacuole lumen. Vacuolar hydrolases eventually degrade or process the sequestered cargo (Feng et al. 2014).

Autophagy initiates when two protein complexes are triggered at phagophore assembly sites, UN51-like Ser/Thr kinase (ULK) and phosphatidylinositol-3-kinase (PI3K) (Agarwal et al. 2015; Ohsumi and Mizushima 2004). ULK complexes consist of the ULK1/2 and FAK family interacted proteins, and autophagy-related gene 13 (ATG13) (Jung et al. 2009). The PI3K complex comprises Vps34, Vps15, Beclin1, and ATG14 (Fan et al. 2011). It is noteworthy that anti-apoptotic dimers BCL-XL and BCL-2 regulate Beclin1 localized on endoplasmic reticulum (ER) membranes. Beclin1 is dissociated from BCL-2. Next, it coordinates with Vps34 after autophagy is triggered (Marquez and Xu 2012; Martyniszyn et al. 2011; Pattingre et al. 2005). As a result, phosphatidylinositol 3-phosphate (PI3P) will concentrate on the phagophore’s surface (Obara and Ohsumi 2008; Puri et al. 2013). Autophagosome expansion and closure are mediated via two ubiquitin-like complexes. Initially, upon the interaction of Atg7, Atg5 covalently attaches to Atg12 (Shao et al. 2007). The complex then binds to Atg16 for Atg5-Atg12-Atg16 complex formation, which is accountable for phagophore elongation. Atg4B cleaves microtubule-associated protein 1 light chain 3 (LC3) to form LC3-I, in another ubiquitin-like complex (Fujita et al. 2008). Next, LC3-I is converted to phosphatidylethanolamine (PE)-conjugated LC3-II with assistance of the Atg5-Atg12-Atg16 complex. LC3-II is considered an important marker for autophagosomes (Kabeya et al. 2000). Subsequently, mature autophagosomes move along microtubules to fuse with lysosomes (Ravikumar et al. 2005). This involves the recruitment of multiple membrane protein complexes such as soluble NSF attachment protein receptors (SNAREs) (Itakura et al. 2012). A proteolytic reaction occurs after autolysosomes are formed to degrade the cargoes they carry (Guo et al. 2018b).

The pathophysiology of AD includes two abnormal structures: Neurofibrillary tangles (NTs) and senile plaques. Altered cleavage of amyloid precursor protein (APP) has been implicated in increased β-amyloid tangles, constituting senile plaques (Querfurth and LaFerla 2010). NTs, which are hyperphosphorylated tau proteins associated with microtubules, are pathological, insoluble aggregates. In normal conditions, microtubule stabilization and vesicle transport in neurons are controlled by tau’s interaction with tubulin (Wang et al. 2013). AD brains with autophagic vacuoles (AVs) are indicative of altered autophagy in this disease. Disruption of retrograde transport of autophagosomes along axons results in the repletion of naive AVs and Aβ-producing AVs (Ułamek-Kozioł et al. 2013; Yu et al. 2005), leading to increased production of Aβ. Autophagy contributes to clearance of soluble as well as insoluble tau aggregates in conjunction with the ubiquitin-proteasome system. Chloroquine, causes tau clearance to be delayed and tau aggregates to accumulate by inhibiting autophagosome–lysosome fusion (Hamano et al. 2008). Phosphorylated tau is more affected by autophagic failure than other forms of tau (Rodríguez-Martín et al. 2013).

Another common neurodegenerative disorder is PD, characterized by accumulation of α-synuclein and degeneration of dopaminergic neurons, and presence of ubiquitin in Lewy bodies, which are intracytoplasmic inclusions (Tan et al. 2014).

**Fig. 1:**
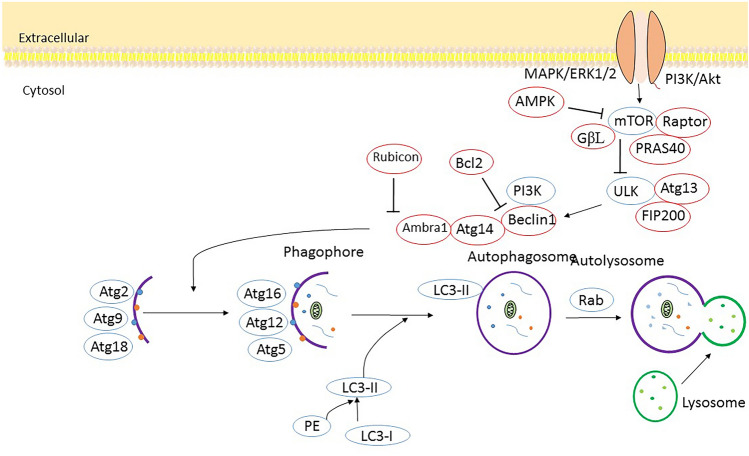
A schematic representation of the autophagy pathway. This figure adapted from (Riebisch et al. 2021)

Autophagic dysfunction in the substantia nigra is inherent to PD (Alcalay et al. 2010). Autophagy pathway can be regulated by proteins encoded by PD-relevant genes. LRRK2, one of the genes whose mutation is associated with PD, may regulate macroautophagy (Bonifati 2006). Knockdown of LRRK2 stimulates autophagy and inhibits LRRK2 kinase activity, thus increasing macroautophagy, absent changes in TORC1 levels (Alegre-Abarrategui et al. 2009; Manzoni et al. 2013). Mitophagy also mediates the function of autosomal recessive genes associated with PD. Parkin, an E3-like ligase, mostly localizes to the cytosol, but once a mitochondrial uncoupler (CCCP) is applied, it is translocated to damaged mitochondria for removal (Narendra et al. 2008). As a result, Parkin mutations block the ability of mitophagy to clear damaged mitochondria in PD. In addition, studies have shown that depolarization of mitochondria recruits Parkin by accumulating PINK1 on its external membrane (Scarffe et al. 2014). Nonetheless, the link between mitophagy and the etiology of PD has yet to be fully characterized. Moreover, oxidative stress has been shown to trigger PD. In this regard, autophagy, which reduces oxidative stress, may be an effective treatment for PD (Surendran and Rajasankar 2010). The PD SNCA model of the BECN1 gene showed attenuation in neurodegenerative pathology (Spencer et al. 2009). Overexpressing RAB1A, which controls cell membrane transfer and autophagosome construct, alleviates deficiencies in dopaminergic neurons expressing SNCA (Coune et al. 2011). Dopaminergic neurodegeneration by SNCA in *Drosophila* has been shown to be inhibited by histone deacetylase 6, an enzyme controlling autophagosomal maturation (Du et al. 2010). Enhanced expression of transcription factor EB leads to clearance of SNCA from dopaminergic neurons through autophagy (Decressac et al. 2013). PD patients’ brains have lower expression of LAMP2A and HSC70, the constituents of CMA (Sala et al. 2014). Taken together, these studies show that autophagy has an important function in the pathogenesis of PD (Fig. 1).

Microglia, the brain’s resident macrophages with an inherent ability to respond to CNS injury, play a crucial role in promoting repair and ensuring proper brain function. While studies examining the impact of autophagy on neurodegenerative disorders has concentrated on neurons, recent findings suggest that autophagy might also play a role in the functioning of glial cells (Dello Russo et al. 2013; Yamamoto and Yue 2014). Increased evidence purports that autophagy is regulated by both innate and adaptive responses in the peripheral immune system (Shibutani et al. 2015) and the process of phagocytosis (Green et al. 2016). Microglia are the brain’s phagocytes (Sierra et al. 2013). Autophagy and phagocytosis share striking morphological and mechanistic similarities, as both processes rely on the formation of transient vesicular structures (autophagosomes and phagosomes, respectively) that engulf and deliver cargo to the lysosomes for digestion. Interestingly, a functional cross-talk exists between autophagy and phagocytosis during the innate immune response in peripheral macrophages. The modulation of autophagy in microglia could affect both microglial phagocytosis and inflammation, potentially playing a role in the progression of neurodegeneration (Plaza-Zabala et al. 2017). Microglial autophagy has the potential to affect the activation and inflammatory responses in microglia (Su et al. 2016). In one study, the inhibition of autophagy was shown to decrease Aβ phagocytosis (Lucin et al. 2013). Microglial autophagy increased Aβ degradation and NLRP3 inflammasome in AD (Cho et al. 2014). Furthermore, the disruption of microglia autophagy exacerbated neuroinflammation and dopaminergic neuron loss via targeting NLRP3 inflammasome in animal model of PD induced by MPTP (Cheng et al. 2020; Qin et al. 2021b). Therefore, microglia autophagy plays a neuroprotective role in the progression of AD and PD.

## Non-coding RNAs

A series of cleavage events produce mature microRNAs after RNA polymerases II and III transcribe microRNA precursors (Annese et al. 2020). In the canonical pathway of biogenesis, they are processed into pre-miRNAs by the microprocessor complex, including a ribonuclease III enzyme, Drosha, and an RNA binding protein DiGeorge Syndrome Critical Region 8 (DGCR8) (Gregory et al. 2004). In addition, there are two main non-canonical miRNA biogenesis pathways: Dicer-independent and Drosha/DGCR8-independent (Felekkis et al. 2010).

miRNAs expression in neurodegenerative diseases is altered, providing further evidence for their putative function in neurodegeneration. For instance, transfection of miR-7 suppresses microglial NLRP3 inflammasome activation and decreased dopaminergic neuron degeneration by decreasing microglial activation in the PD model induced by MPTP (Titze-de-Almeida and Titze-de-Almeida 2018). miR-7 inhibited neuronal apoptosis in PD cell lines model via targeting Sirt2 and Bax (Li et al. 2016). However, the upregulation of miR-7 promoted the production of extracellular Aβ in nerve cells (Fernández-de Frutos et al. 2019). miR-106b enhanced levels of secreted Aβ by regulating ABCA1 (Kim et al. 2012). miR-106b affected TGF-β signaling, thereby contributing to the pathogenesis of AD (Wang et al. 2010). Zhang et al. (2022) demonstrated that miRNA-mediated autophagy might be a putative therapeutic strategy AD.

lncRNAs also contain a poly (A) terminus at 3′ and a 5′ methyl-cytosine cap (Zhang et al. 2019), which are transcribed by RNA polymerase II (Pol II) (Nojima and Proudfoot 2022). lncRNAs biogenesis is analogous to that of mRNA with few variations. There is strong evidence that lncRNAs are capped and polyadenylated by canonical splicing in the vast majority of cases (Gourvest et al. 2019).

Information on LncRNAs target genes and signaling pathways in neurodegenerative diseases has been recently addressed. lnc-ANRIL knockdown inhibits cell inflammation via the binding of miR-125a in vitro model of AD (Zhou et al. 2020a). LncRNA H19 decreases apoptosis in neurons of MPTP-induced PD mice via regulating miR-585-3p/PIK3R3 (Zhang et al. 2020b). LncRNA-mediated autophagy may be a strategy for the treatment of neurodegenerative diseases (Jiang and Xu 2023; Xu et al. 2020).

RNA circular molecules (circRNAs) are endogenous non-coding RNA generated by the back-splicing process. Some circRNAs consisting of introns originate in the nucleus, while others have one or more exons with major locations in the cytoplasm (Guo et al. 2014). A circular RNA does not have a polyadenylated tail or a 5–3′ direction, rendering them more stable in plasma and tissues as they have a continuous loop of covalent bonds (Guo et al. 2014; Suzuki and Tsukahara 2014). For instance, circDLGAP4 has neuroprotective effects in animal models of PD by targeting the miR-134-5p and CREB (Feng et al. 2020). Circular RNA Cwc27 participates in the pathogenesis of AD by repressing Pur-α activity (Song et al. 2022).

## Non-coding RNAs and Autophagy in AD and PD

### miRNAs and Autophagy in AD and PD

#### miR-7 and miR-153

An in vitro study showed that miR-7 or miR-153 overexpression protected cortical neurons against MPP +. A small amount of miR-153 debilitated MPP + -induced initiation of p38MAPK, while miR-7 preserved that of mTOR, JNK , and SAPK protein expressions. Rapamycin co-administered with MPP + ameliorated the neuroprotective influence of miR-7 and miR-153 (Fragkouli and Doxakis 2014). An in vitro study showed that miR-7 reduced α-Syn expression and degraded α-Syn without targeting the 3′-UTR of the α-Syn mRNA. Its function is applied by increasing autophagy via the conversion of LC3-I to LC3-II and autophagosome formation (Choi et al. 2018).

#### miR-9-5p

miR-9-5p exacerbates MPP +—induced neurotoxicity by targeting SIRT1 (Wang et al. 2019). miR-9-5p antagomirs increased Aβ clearance and improved cognition in APPswe/PS1dE9 mice by targeting autophagy via autophagy receptor Optineurin (Optn) (Chen et al. 2021).

#### miR-29c

A PD mouse model has shown enhanced autophagy and down-regulation of miR-29c-3p in dopamine neurons. In a PD mice and in vitro models, increased miR-29c-3p reduced autophagy by decreasing LC3-II/I and Beclin-1 and increasing p62 expression via targeting of TET2 (Wang et al. 2021a).

#### miR-101

miR-101 is implicated in normal cognitive function. It has a vital role in the pathology of aging-related neurodegenerative diseases (Barbato et al. 2020). MiR-101 decreased APP in AD (Long and Lahiri 2011). Increased miR-101 led to α-syn accumulation (Valera et al. 2017). miR-101 mimic inhibited cell death in PD (Omura et al. 2021). miRNA-101a markedly down-regulated in AD patients and mouse. miRNA-101a transfection decreased autophagy in in vitro model of AD by targeting MAPK1 pathway, beclin-1 (Li et al. 2019).

#### miR-124

miR-124 is known as a neuron-specific miR (Lagos-Quintana et al. 2002; Mishima et al. 2007). The increased miR-124 level may be effective in decreasing neuroinflammation (Han et al. 2020). Exosomal miR-124-3p transferring into hippocampal neurons decreased neuronal injury by regulating the Rela and ApoE expressions (Ge et al. 2020). Translation of miR-124 in APP/PS1 transgenic mice showed improvements in their AD pathology and learning ability. As a consequence, the expression of p62/SQSTMl, Atg5, and LC3II was down-regulated, while Beclin-1 levels were increased (Du et al. 2017). Upon targeting LC3II/I expression, overexpression of miR-124 increased autophagy in PD induced by MPTP (Yao et al. 2019). Moreover, miR-124 was suppressed in SK-N-SH cell line treated with MPTP, resulting in enhanced autophagy by increased LC3-II/I and Beclin-1. In addition, p-AMPK levels increased in neurons upon miR-124 suppression, while p-mTOR levels decreased (Gong et al. 2016). Increased miR-124 led to decreased loss of dopamine by regulating apoptosis and autophagy in animal model of PD induced by MPTP. miR-124 decreased autophagosome accumulation and lysosomal depletion in PD (Wang et al. 2016).

#### miR-132/212

Tauopathies such as AD are associated with reduced microRNA (miRNA) cluster expression, miR-132/212 (Wang et al. 2017b). Decreased miR-132/212 expressions led to AD pathogenesis (Wang et al. 2017b). An in vivo study showed that a deficiency of miR-132/212 results in increased tau, along with increased tau aggregation. miR-132 directly regulated tau expression. Induction of tau aggregation by miR-132/212 deletion was associated with dysfunctional autophagy via targeting Beclin-1, ATG5–12, 9a, P62 and TMEM106b. On the other hand, miR-132 mimics were shown to restore in part cognition and tau expression in AD mice treated with miR-132 mimics. Finally, miR-132 and miR-212 were shown to be associated with cognitive dysfunction and insoluble tau in humans (Smith et al. 2015).

#### miR-133a

In cells treated with MPP + , a model for PD, miR-133a expression levels decreased. MiR-133a overexpression enhanced cell proliferation and autophagy (LC3II/I and Beclin-1 expression declined, while p62 expression increased), but inhibited apoptosis. The inhibition of PC12 cell autophagy and apoptosis by miR-133a was attenuated by RACY upregulation by miR-133a targeting RAC1 (Lu et al. 2020).

#### miR-181

Dysregulation of miR-181a is inherent to neurodegenerative diseases (Hegarty et al. 2018). miR-181a is upregulated in patients with MCI. It is involved in AD pathogenesis (Ansari et al. 2019). The inhibition of miR-181a improved synapse and cognitive function in 3xTg-AD animals (Rodriguez‐Ortiz et al. 2020). However, miR-181a was decreased in vitro model of PD (Liu et al. 2017). miR-181a/b suppressed synaptic transmission, mitochondrial respiration, and neurite outgrowth-related gene expression in MPP + -treated SK-N-SH cells (Stein et al. 2022). The upregulation of miR-181a inhibited autophagy by targeting Beclin-1, LC3II/I, p-p38, and p-JNK (Liu et al. 2017). In addition, miR-181b inhibited MPP + -induced cytotoxicity in PC12 cells via the inhibition of autophagy by targeting PTEN, Akt and mTOR expression (Li et al. 2018).

#### miR-185

The protective function of miR-185 was shown in controlling PD progression (Rahimmi et al. 2019). miR-185 decreased cell death in dopaminergic neuron by activating PI3K/AKT signaling in PD (Qin et al. 2021a). Increased miR-185 suppressed autophagy in cells treated with MPTP by targeting the AMPK and mTOR (Wen et al. 2018).

#### miR-204

miR-204 is upregulated in neurodegenerative diseases (Talepoor Ardakani et al. 2019). Enhancement in miR-204 expression was observed in AD (Zhang et al. 2021a). The upregulation of miR-204-5p induced dopaminergic cell death by targeting ER stress-mediated by DYRK1A and apoptosis (Chiu et al. 2019). Overexpression of TRPML1 led to downregulation of miR-204 and activation of STAT3, while mitochondrial autophagy was attenuated. miR-204 silencing suppressed mitochondrial autophagy by inhibiting Parkin, PINK1,,LC3II, and Beclin1 through STAT3 pathway and up-regulating TRPML1 expression in APP/PS1 transgenic AD mice and a cell model of AD induced by Aβ1-42 (Zhang et al. 2021a).

#### miR-214

Decreased miR-214 contributes to the neuronal injury induced by Aβ (Yu and Zhang 2013). MiR-214–5p expression decreased in AD mice. Dexmedetomidine has a neuroprotective effect on AD via targeting miR-214–5p (Hu et al. 2022). miR-214-3p inhibited autophagy by inhibiting Atg12 in SAMP8 mice (Zhang et al. 2016a).

#### miR-221/222

In MN9D cells treated with MPP +, miR-221/222 was targeted and bound to p27/mTOR. By downregulating p27 levels, inhibiting the mTOR pathway. Thus, miR-221/222 facilitates autophagy and protects cells from death (Qian et al. 2019). MiR-212-5p protected against cell death in dopaminergic neuron by targeting p53, SIRT2, LC3 B and p62 expression in an animal model of PD induced by MPTP (Sun et al. 2018).

The number of miRNAs associated with autophagy in AD and PD is also continually expanding as shown in Table 1.

**Table 1:** Autophagy-related microRNAs in AD and PD

Neurodegenerative disease	Model	miRNAs	Expression status	Targets	Main targets	References
Alzheimer disease	In vitro/SH-SY5Y cells treated by H_2_O_2_	miR-101a	Downregulated	MAPK1 pathway, beclin-1, LC3-II	Stimulate autophagy	Li et al. (2019)
Alzheimer disease	In vitro/PC12 and SK-N-SH cells	let-7	Upregulated	Atg-5, -7, LC3 II/I, beclin-1, PI3K/Akt/mTOR	Stimulate autophagy	Gu et al. (2017)
Alzheimer disease	In vitro/cycloheximide-treated SH-SY5Y cells	miR-9a	Upregulated	*UBE4A* and *UBE4B*	Stimulate autophagy	Subramanian et al. (2021)
	In vivo/E64D + PEPA-treated *Tau-BiFC* mice					
Alzheimer disease	in vivo/SAMP8 mice	miR-214-3p	Downregulated	Atg12, 3′-UTR, LC3βII and Beclin1	Inhibit autophagy	Zhang et al. (2016a)
Alzheimer disease	In vivo/APPswe/PS1dE9 mice	miR-299-5p	Downregulated	LC3βII, p62 and Atg5	Inhibit autophagy	Zhang et al. (2016b)
	In vitro/N2a/SH-SY5Y cells					
Alzheimer disease	In vivo/SH-SY5Y cell APPswe/PS1dE9 mouse	miR-9-5p and miR-331-3p	Downregulated	Aβ clearance, *Sqstm1, Optn*, *LC3b* and* Becn1*	Stimulate autophagy	Chen et al. (2021)
Alzheimer disease	In vivo/APP/PS1 transgenic mice	miR-124	Downregulated	BACE1, LC3II, Atg5, p62/SQSTMl, Beclin-1	Stimulate autophagy	Du et al. (2017)
Alzheimer disease	In vivo/3xTg-AD^WT^ mice	miR-132/212	Downregulated	tau mRNA, ATG5–12, ATG9a, Beclin1, P62, TMEM106b	Inhibit autophagy	Smith et al. (2015)
Alzheimer disease	In vivo/Aβ1-42 and APP/presenilin-1 AD modeled mice	miR-204	Upregulated	TRPML1 -activated STAT3 pathway	Inhibit autophagy	Zhang et al. (2021a)
Parkinson disease	In vitro*/*SK-N-SH neuroblastoma cells treated by MPP	miR-181a	downregulated	LC3II/LC3I, Beclin 1, p-p38, p-JNK	Stimulate autophagy	Liu et al. (2017)
Parkinson disease	In vivo/SH-SY5Y cells, BV2 cells MPTP-treated, treated with VX702	miR-124	downregulated	p62, p-p38	Inhibit autophagy	Yao et al. (2019)
Parkinson disease	in vitro/mouse microglia cell line (BV2) treated by MPTP	miR-3473b	upregulated	TNF-α, IL-1β, TREM2 and ULK1	Inhibit autophagy	Lv et al. (2021)
Parkinson disease	in vivo/C57BL/6 mice model treated by MPTP	miR-3473b	upregulated	substantia nigra pars compacta (SNpc), TREM2, ULK1	Inhibit autophagy	Lv et al. (2021)
Parkinson disease	In vivo/SH-SY5Y neuroblastoma cells MPTP-treated	miR-212-5p	downregulated	SIRT2, P53	Stimulate autophagy	Sun et al. (2018)
	In vitro/MPP^+^ SH-SY5Y cells					
Parkinson disease	In vivo/C57BL/6 mice HEK‐293T MPTP/MPP + ‐induced	miR-326	upregulated	JNK, XBP1, c-Jun, p–c-Jun, α-Syn, p-α-Syn, iNOS and LC3-II	Stimulate autophagy	Zhao et al. (2019)
Parkinson disease	In vitro/HEK293A cells MPP^+^	miR-7 and miR-153	Upregulated	mTOR, SAPK/JNK, P38MAPK, Bcl-2, Caspase-3, LC3-I, LC3-II	Inhibit autophagy	Fragkouli and Doxakis (2014)
Parkinson disease	In vitro/SH-SY5Y cells treated by MPTP	miR-124	Downregulated	Beclin 1, LC3 II/I, AMPK, mTOR	Stimulate autophagy	Gong et al. (2016)
Parkinson disease	In vivo/MPTP-treated mice	miR-221/222	Upregulated	p27/mTOR, LC3-II, (CDKN1B/p27)	Inhibit autophagy	Qian et al. (2019)
	In vitro/MPP + -challenged MN9D cells					
Parkinson disease	In vitro/MPTP-treated SH-SY5Y cells	miR-185	Upregulated	AMPK, mTOR	Inhibit autophagy	Wen et al. (2018)
Parkinson disease	In vivo/Bcl-2 treated 6-OHDA-induced	miR-3557	Upregulated	CaMK2α, CaMKV, Vdac1, PI3K/mTOR and UCH-L1		Liu et al., (2019)
Parkinson disease	In vivo/SH-SY5Y cells were treated with MPP^+^	miR-29c-3p	Downregulated	Beclin 1, TET2, LC3	inhibits autophagy	Wang et al. (2021a)
Parkinson disease	In vivo/MPTP-treated mice	miR-124	Upregulated	Bim, Bax	Inhibit autophagy	Wang et al. (2016)
	In vitro/MPP^+^-intoxicated SH-SY5Y cells					
Parkinson disease	In vitro/PC12 cells MPP^+^	miR-199a	Upregulated	PTEN, GSK3β, Beclin1, LC3II, AKT, mTOR	Inhibit autophagy	Ba et al. (2020)
Parkinson disease	In vitro/MPP^+^ treated PC12 cell	miR-181b	Downregulated	LC3II, p-AKT, PTEN, p-mTOR, p-p70S6K	Inhibit autophagy	Li et al. (2018)
Parkinson disease	In vivo/MPTP	miR-106b	Downregulated	Bcl-2 and LC3II/LC3I ratio	Inhibit autophagy	Bai et al. (2021)
Parkinson disease	In vitro/Human neural progenitor cell line ReNcell V	miR-7	Downregulated	LC3-I/LC3-II	Inhibit autophagy	Choi et al. (2018)
Parkinson disease	SH-SY5Y cells treated with MPP^+^	MiR-497-5p	Upregulated	LC3-II/I, Beclin1, p62	Inhibit autophagy	Zhu et al. (2021)
Parkinson disease	In vitro/PC-12 treated by MPP^+^	miR-133a	Downregulated	LC3II/I, Beclin-1, p62	Stimulate autophagy	Lu et al. (2020)
Parkinson disease	MPP^+^- treated SH-SY5Y cells	miR-132-5p	Upregulated	LC3, Beclin 1, ULK1	Stimulate autophagy	Zhao et al. (2020c)
Parkinson disease	In vitro/SH-SY5Y cells	miR-103a-3p	Upregulated	Parkin,Ambra1, LC3-I/LC3-II	Stimulate autophagy	Zhou et al. (2020b)
	In vivo/MPTP					
Parkinson disease	6-OHDA-treated SH-SY5Y cell model	MiR-142-5p	Downregulated	decreased P62, increased LC3-II, Beclin-1	Stimulate autophagy	Chen et al. (2020)
Parkinson disease	In vivo/rotenone	MiR-24	Upregulated	Beclin-1, LC3-II/LC3-I ratio	Inhibit autophagy	Ge et al. (2019)
	In vitro/MN9D cells					

### lncRNAs and Apoptosis in AD and PD

#### BACE1-AS

Increased BACE1-AS expression is inherent to AD (Zhou et al. 2021). BACE1-AS downregulation was related to decreased synuclein, iNOS and glutamate (Li et al. 2020). ATG5 expression was indirectly regulated by BACE1-AS through the interaction with miR-214-3p. Neuronal damage secondary to inhibition of autophagy is relieved by suppression of BACE1-AS in vivo (Zhou et al. 2021). In AD, it has been shown both in vitro and in vivo that BACE1-AS and miR-214-3p are overexpressed and suppressed, respectively. BACE1-AS reduction as well as miR-214-3p overexpression promote the acceleration of autophagy by targeting LC3 I/II, p62 and Beclin-1 (He et al. 2020).

#### NEAT1

Increased NEAT1 expression was noted in SH-SY5Y cells upon MPP + treatment. NEAT1 silencing restored the MPP + -induced effects on SH-SY5Y cells, including inflammation, cell viability, and stimulated apoptosis. NEAT1 targets miR-124 and targeting miR-124 with anti-miR-124 reversed its inhibition effects (Xie et al. 2019). NEAT1 level was elevated in vitro AD experimental model, while its depletion reversed Aβ-induced increase in apoptosis and p-Tau levels. MiR-107 is targeted by NEAT1. miR-107 expression decreased in Aβ-treated cells, and its upregulation reversed Aβ-induced damage. Neuronal injury in Aβ-treated cells was suppressed by NEAT1 knockdown and reversed by miR-107 (Ke et al. 2019). Animal models of AD have shown upregulation of NEAT1 lncRNA. Inhibition of PINK1-dependent autophagy by ubiquitinating and degrading PINK1 has been posited as a potential sequela of NEAT1 interaction with NEDD4L (Huang et al. 2020). A significant increase in NEAT1 was observed in MPTP-treated mice and PD patients, as well as in SH-SY5Y cells treated with MPP + (Dong et al. 2021; Liu et al. 2020; Xie et al. 2019). SH-SY5Y cells treated with MPP + increased proliferation upon NEAT1 inhibition, whereas apoptosis and autophagy were inhibited. MPTP-treated mice with NEAT1 inhibition showed enhanced MIR-374c-5p expression, increased cell viability, and repressed autophagy and apoptosis by targeting miR-374c-5p, LC3 II/LC3 I, P62 (Dong et al. 2021). Autophagy was suppressed in in vitro and in vivo model of MPTP- induced PD by NEAT1 knockdown via targeting PINK1, LC3-II, and LC3-I protein, decreasing dopaminergic neuronal damage (Yan et al. 2018).

#### SNHG1

MPP + enhanced expression of SNHG1 (Zhang et al. 2020a; Zhao et al. 2020a). Furthermore, silencing SNHG1 improved the behavior in MPTP-treated mice in vivo (Xiao et al. 2021). In addition to promoting autophagy and preventing MPP + -induced cell death, SNHG1 indirectly affects the expression of p27/mTOR by binding to the miR-221/222 cluster (Qian et al. 2019).

#### HOTAIR

SH-SY5Y cells treated with MPP + showed enhanced expression of lncRNA HOTAIR. PD-like symptoms were significantly alleviated in vivo when HOTAIR was knocked down. As a result of the downregulation of HOTAIR in the presence of MPP +, SH-SY5Y cells increased their viability and NLRP3-mediated pyroptotic cell death was suppressed. By targeting miR-326, HOTAIR regulates ELAVL1 expression. Downregulation of HOTAIR or ELAVL1 significantly reduced the pyroptosis-promoting effects of miR-326 inhibitor through activation of NLRP3 inflammasomes (Zhang et al. 2021c). In SK-N-SH cells treated with MPP + , increased expression of HOTAIR and ATG10 was noted (Zhao et al. 2020b). MPP + -induced neurodegeneration was decreased by the knockdown of HOTAIR. HOTAIR aggravates MPP + -mediated neuronal injury by sponging miR-874-5p (Zhao et al. 2020b). MTPT-treated mice and SH-SY5Y cells pretreated with MPP + showed upregulation of HOTAIR. In SH-SY5Y cells overexpressing HOTAIR, the expression of LRRK2 was enhanced. In SH-SY5Y cells treated by MPP +, HOTAIR knockdown improved cell viability (Kraus et al. 2017; Wang et al. 2017a). HOTAIR inhibited cell viability and stimulated autophagy by attaching to miR-221-3p and targeting LC3-II/-I, LAMP1/2, P62 expressions, and NPTX2 (Lang et al. 2020).

#### BDNF-AS

BDNF-AS expression increased in AD patients. The upregulation of BDNF-AS led to cognitive dysfunction in AD mice (Ding et al. 2022). Silencing BDNF-AS inhibited apoptosis and oxidative stress in PC12 cells treated by Aβ25-35 (Guo et al. 2018a). Fan et al. (Fan et al. 2020) was seen the up-regulation of BDNF-AS in both in vitro and in vivo model of PD. BDNF-AS increased autophagy in MPTP-induced PD by targeting microRNA-125b-5p, LC3II/I, and Beclin-1, p62 levels (Fan et al. 2020).

The number of lncRNAs associated with autophagy in AD and PD is also continually expanding as shown in Table 2.

**Table 2:** Autophagy-related lncRNAs in AD and PD

Neurodegenerative disease	Model	lncRNAs	Expression status	Targets	Main effect	References
Alzheimer disease	In vivo/transgenic mice,	BACE1-AS	Upregulated	miR-214-3p and ATG5	Stimulate autophagy	Zhou et al. (2021)
	In vitro/SH-SY5Y treated by Aβ_1-42_					
Alzheimer disease	In vitro/Aβ-treated SK-N-SH and SK-N-AS cells	BACE1-AS	Upregulated	miR-214-3p, Bax, Bcl-2, Beclin-1, LC3 I/LC3 II and p62	Stimulate autophagy	He et al. (2020)
	In vivo					
Alzheimer disease	In vivo/transgenic mice	lncRNA *RMRP*	Upregulated	*miR-3142*/*TRIB3* axis	Stimulate autophagy	Tang et al. (2022)
	In vitro/SH-SY5Y cells					
Parkinson disease	MPP^+^	lncRNA-SNHG1	Upregulated	miR-221/222 cluster, CDKN1B/p27/mTOR, SNHG1	inhibit autophagy	Qian et al. (2019)
Parkinson disease	In vivo	NEAT1	Upregulated	PINK1, LC3-I, LC3-II	Stimulate autophagy	Yan et al. (2018)
	In vitro/MPP + treated-SH-SY5Y cells					
Parkinson disease	In vitro/MPP +—treated SH-SY5Y cells	NEAT1	Upregulated	miR-374c-5p, LC3 II/LC3 I, P62	Stimulate autophagy	Dong et al. (2021)
	In vivo/MPTP					
Parkinson disease	In vitro/MN9D was treated (MPP^+^), In vivo/MPTP	HOTAIR	Downregulated	LC3B-II/LC3B-I, LAMP1/LAMP2, P62, NPTX2	Inhibit autophagy	Lang et al. (2020)
Parkinson disease	In vivo/MPTP	BDNF-AS	Upregulated	miR-125b-5p, LC3II/I and Beclin-1, p62 levels	Inhibit autophagy	Fan et al. (2020)
	In vitro/MPP +					

### Circular RNAs and Apoptosis in AD and PD

Autophagy is regulated by CircumNF1-419 via Akt-mTOR/PI3K-I and Akt-AMPK-mTOR/PI3K-I signaling pathways. AP2B1 injection into circNF1-419 overexpressing cerebral cortex increased autophagy. Consequently, tau and p-tau were decreased, suggesting a delay in the onset of senile dementia. CirNF1-419 enhanced the activity of several signaling pathways, particularly mediators of transmission in the SAMP8 mouse (Diling et al. 2019). In in vitro model of PD, expression levels of SNCCa and circSNCA were downregulated after PPX treatment, which correlated with apoptotic gene expression, and SNCA expression was increased by CircSNCA through the downregulation of miR-7 in PD as a competitive endogenous RNA (ceRNA). Pro-apoptotic proteins were less expressed in cells with lower circSNCA expression. Downregulation of CircSNCA in PD reduced apoptosis and promoted autophagy (Sang et al. 2018). circDLGAP4 expression decreased in PD mouse model inculcated with MPTP. circDLGAP4 overexpression mitigated mitochondrial impairment, and enhanced cell viability and autophagy, decreased apoptosis and thereby attenuated neurotoxicity. A major function of circDLGAP4 is to regulate miR-134-5p. MiR-134-5p targets the transcription factor CREB, and CREB expression can be affected by the circDLGAP4/miR-134-5p axis. CircDLGAP4/miR-134-5p can also modulate the activity of CREB signaling, PGC-1α, Bcl-2, and BDNF (Feng et al. 2020). circSAMD4A was upregulated in both PD animal and cellular models. Inhibition of MPTP or MPP +-induced apoptosis and autophagy by circSAMD4A knockdown was observed. In MPTP or MPP +-induced PD models, the knockdown of circSAMD4A resulted in a reduction in phosphorylated AMPK expression and a subsequent increase in mTOR expression (Wang et al. 2021b) (Table 3).

**Table 3:** Autophagy-related lncRNAs in AD and PD

Neurodegenerative disease	Model	CircRNAs	Expression status	Targets	Main effect	References
Alzheimer disease	In vivo/SAMP8 mice treated with d-galactose	*circNF1-419*	Upregulated	Dynamin-1, AP2B1, PI3K-I, Akt, AMPK, mTOR, Aβ_1-42,_ Tau, p-Tau, and APOE	Stimulate autophagy	Diling et al. (2019)
Parkinson disease	In vitro/(MPP +) SH-SY5Y cells	CircSNCA	Downregulated	miR-7 sponge to upregulate SNCA	Induce autophagy	Sang et al. (2018)
Parkinson disease	In vivo/MPTP	circDLGAP4	Downregulated	miR-134-5p/CREB, Beclin-1, LC3 II/I ratio	Inhibit autophagy	Feng et al. (2020)
	In vitro/SH-SY5Y and MN9D cells treated with MPP^+^					
Parkinson disease	In vitro/SH-SY5Y cells MPTP- or MPP^+^	circSAMD4A	Upregulated	AMPK, mTOR, miR-29c-3p	Stimulate autophagy	Wang et al. (2021b)

Finally, dysregulation of circNF1-419, circSNCA, circHIPK3, circDLGAP4, circSAMD4A, circNF1-419, circSHOC2, circERCC2 has been associated with inhibition or stimulation of autophagy in AD and PD.

## Conclusions

Increased evidence suggests that ncRNAs play a key role in autophagic homeostasis and can regulate neurodegeneration in AD and PD. ncRNAs can form complex interactions with other RNAs/DNA/proteins to regulate the autophagic process. For future perspective, the interaction between ncRNAs and autophagy in other neurodegenerative diseases and further clinical studies on the role of ncRNAs in neurodegenerative diseases progression and autophagy are required. In addition, further characterization of ncRNAs in bodily fluids is necessary, in particular, blood and CSF in patients with neurodegenerative diseases. ncRNAs (miRNAs, lncRNAs, and circRNAs) may serve as putative treatment, prognostic, and diagnostic targets for neurodegenerative diseases**.** The development of reliable for diagnosing early neurodegeneration and its molecular signaling will be pivotal for increased efficiency of therapies. Some studies evaluated targeting and transcriptionally repressing ncRNAs by silencing ncRNAs and RNA interference to inhibit ncRNAs expression in neurodegenerative disorders (Wang et al. 2022; Zhang et al. 2021b). Moreover, CRISPRs could be applied to delete miRNAs/lncRNAs (Ho et al. 2015) and assess the consequences for therapeutic strategies.

## Data Availability

Not applicable.

## References

[CR1] Agarwal S, Tiwari SK, Seth B, Yadav A, Singh A, Mudawal A, Chauhan LKS, Gupta SK, Choubey V, Tripathi A (2015) Activation of autophagic flux against xenoestrogen bisphenol-A-induced hippocampal neurodegeneration via AMP kinase (AMPK)/mammalian target of rapamycin (mTOR) pathways. J Biol Chem 290:21163–21184 26139607 10.1074/jbc.M115.648998 PMC4543672

[CR2] Alcalay RN, Caccappolo E, Mejia-Santana H, Tang MX, Rosado L, Ross BM, Verbitsky M, Kisselev S, Louis ED, Comella C (2010) Frequency of known mutations in early-onset Parkinson disease: implication for genetic counseling: the consortium on risk for early onset Parkinson disease study. Arch Neurol 67:1116–1122 20837857 10.1001/archneurol.2010.194 PMC3329730

[CR3] Alegre-Abarrategui J, Christian H, Lufino MM, Mutihac R, Venda LL, Ansorge O, Wade-Martins R (2009) LRRK2 regulates autophagic activity and localizes to specific membrane microdomains in a novel human genomic reporter cellular model. Hum Mol Genet 18:4022–4034 19640926 10.1093/hmg/ddp346 PMC2758136

[CR4] Annese T, Tamma R, De Giorgis M, Ribatti D (2020) microRNAs biogenesis, functions and role in tumor angiogenesis. Front Oncol 10:581007 33330058 10.3389/fonc.2020.581007 PMC7729128

[CR5] Ansari A, Maffioletti E, Milanesi E, Marizzoni M, Frisoni GB, Blin O, Richardson JC, Bordet R, Forloni G, Gennarelli M (2019) miR-146a and miR-181a are involved in the progression of mild cognitive impairment to Alzheimer’s disease. Neurobiol Aging 82:102–109 31437718 10.1016/j.neurobiolaging.2019.06.005

[CR6] Ba R-Q, Liu J, Fan X-J, Jin G-L, Huang B-G, Liu M-W, Yang J-S (2020) Effects of miR-199a on autophagy by targeting glycogen synthase kinase 3β to activate PTEN/AKT/mTOR signaling in an MPP+ in vitro model of Parkinson’s disease. Neurol Res 42:308–318 32151238 10.1080/01616412.2020.1726584

[CR7] Bai X, Dong Q, Zhao L, Yao Y, Wang B (2021) microRNA-106b-containing extracellular vesicles affect autophagy of neurons by regulating CDKN2B in Parkinson’s disease. Neurosci Lett 760:136094 34216715 10.1016/j.neulet.2021.136094

[CR8] Barbato C, Giacovazzo G, Albiero F, Scardigli R, Scopa C, Ciotti M, Strimpakos G, Coccurello R, Ruberti F (2020) Cognitive decline and modulation of Alzheimer’s disease-related genes after inhibition of MicroRNA-101 in Mouse hippocampal neurons. Mol Neurobiol 57:3183–3194 32504417 10.1007/s12035-020-01957-8

[CR9] Bonifati V (2006) Parkinson’s disease: the LRRK2-G2019S mutation: opening a novel era in Parkinson’s disease genetics. Euro J Human Genet: EJHG 14:1061 10.1038/sj.ejhg.5201695 16835587

[CR10] Checkoway H, Lundin JI, Kelada SN (2011) Neurodegenerative diseases. IARC Sci Publ 163:407–419 22997874

[CR11] Chen J, Jiang C, Du J, Xie C-L (2020) MiR-142-5p protects against 6-OHDA-induced SH-SY5Y cell injury by downregulating BECN1 and autophagy. Dose Response 18:1559325820907016 32127787 10.1177/1559325820907016 PMC7036514

[CR12] Chen M-L, Hong C-G, Yue T, Li H-M, Duan R, Hu W-B, Cao J, Wang Z-X, Chen C-Y, Hu X-K (2021) Inhibition of miR-331-3p and miR-9-5p ameliorates Alzheimer’s disease by enhancing autophagy. Theranostics 11:2395 33500732 10.7150/thno.47408 PMC7797673

[CR13] Cheng J, Liao Y, Dong Y, Hu H, Yang N, Kong X, Li S, Li X, Guo J, Qin L (2020) Microglial autophagy defect causes parkinson disease-like symptoms by accelerating inflammasome activation in mice. Autophagy 16:2193–2205 32003282 10.1080/15548627.2020.1719723 PMC7751565

[CR14] Chiu C-C, Yeh T-H, Chen R-S, Chen H-C, Huang Y-Z, Weng Y-H, Cheng Y-C, Liu Y-C, Cheng A-J, Lu Y-C (2019) Upregulated expression of microRNA-204-5p leads to the death of dopaminergic cells by targeting DYRK1A-mediated apoptotic signaling cascade. Front Cell Neurosci 13:399 31572127 10.3389/fncel.2019.00399 PMC6753175

[CR15] Cho M-H, Cho K, Kang H-J, Jeon E-Y, Kim H-S, Kwon H-J, Kim H-M, Kim D-H, Yoon S-Y (2014) Autophagy in microglia degrades extracellular β-amyloid fibrils and regulates the NLRP3 inflammasome. Autophagy 10:1761–1775 25126727 10.4161/auto.29647 PMC4198361

[CR16] Choi DC, Yoo M, Kabaria S, Junn E (2018) MicroRNA-7 facilitates the degradation of alpha-synuclein and its aggregates by promoting autophagy. Neurosci Lett 678:118–123 29738845 10.1016/j.neulet.2018.05.009 PMC5990033

[CR17] Coune PG, Bensadoun J-C, Aebischer P, Schneider B (2011) Rab1A over-expression prevents Golgi apparatus fragmentation and partially corrects motor deficits in an alpha-synuclein based rat model of Parkinson’s disease. J Parkinson’s Dis 1:373–387 23939344 10.3233/JPD-2011-11058

[CR18] Decressac M, Mattsson B, Weikop P, Lundblad M, Jakobsson J, Björklund A (2013) TFEB-mediated autophagy rescues midbrain dopamine neurons from α-synuclein toxicity. Proc Natl Acad Sci USA 110:E1817–E1826 23610405 10.1073/pnas.1305623110 PMC3651458

[CR19] Dello Russo C, Lisi L, Feinstein DL, Navarra P (2013) mTOR kinase, a key player in the regulation of glial functions: relevance for the therapy of multiple sclerosis. Glia 61:301–311 23044764 10.1002/glia.22433

[CR20] Diling C, Yinrui G, Longkai Q, Xiaocui T, Yadi L, Xin Y, Guoyan H, Ou S, Tianqiao Y, Dongdong W (2019) Circular RNA NF1-419 enhances autophagy to ameliorate senile dementia by binding dynamin-1 and adaptor protein 2 B1 in AD-like mice. Aging (albany NY) 11:12002 31860870 10.18632/aging.102529 PMC6949063

[CR21] Ding Y, Luan W, Wang Z, Cao Y (2022) LncRNA BDNF-AS as ceRNA regulates the miR-9-5p/BACE1 pathway affecting neurotoxicity in Alzheimer’s disease. Arch Gerontol Geriatr 99:104614 34990931 10.1016/j.archger.2021.104614

[CR22] Dong L, Zheng Y, Gao L, Luo X (2021) lncRNA NEAT1 prompts autophagy and apoptosis in MPTP-induced Parkinson’s disease by impairing miR-374c-5p. Acta Biochim Biophys Sinica. 10.1093/abbs/gmab055 10.1093/abbs/gmab055 33984130

[CR23] Du G, Liu X, Chen X, Song M, Yan Y, Jiao R, Wang C-C (2010) Drosophila histone deacetylase 6 protects dopaminergic neurons against α-synuclein toxicity by promoting inclusion formation. Mol Biol Cell 21:2128–2137 20444973 10.1091/mbc.E10-03-0200 PMC2893978

[CR24] Du X, Huo X, Yang Y, Hu Z, Botchway BO, Jiang Y, Fang M (2017) miR-124 downregulates BACE 1 and alters autophagy in APP/PS1 transgenic mice. Toxicol Lett 280:195–205 28867212 10.1016/j.toxlet.2017.08.082

[CR25] Dugger BN, Dickson DW (2017) Pathology of neurodegenerative diseases. Cold Spring Harb Perspect Biol 9:a028035 28062563 10.1101/cshperspect.a028035 PMC5495060

[CR26] Duve CD, Wattiaux R (1966) Functions of lysosomes. Ann Rev Physiol 28:435–492 5322983 10.1146/annurev.ph.28.030166.002251

[CR27] Esteller M (2011) Non-coding RNAs in human disease. Nat Rev Genet 12:861–874 22094949 10.1038/nrg3074

[CR28] Fan W, Nassiri A, Zhong Q (2011) Autophagosome targeting and membrane curvature sensing by Barkor/Atg14 (L). Proc Natl Acad Sci USA 108:7769–7774 21518905 10.1073/pnas.1016472108 PMC3093500

[CR29] Fan Y, Zhao X, Lu K, Cheng G (2020) LncRNA BDNF-AS promotes autophagy and apoptosis in MPTP-induced Parkinson’s disease via ablating microRNA-125b-5p. Brain Res Bull 157:119–127 32057951 10.1016/j.brainresbull.2020.02.003

[CR30] Felekkis K, Touvana E, Stefanou C, Deltas C (2010) microRNAs: a newly described class of encoded molecules that play a role in health and disease. Hippokratia 14:236 21311629 PMC3031315

[CR31] Feng Y, He D, Yao Z, Klionsky DJ (2014) The machinery of macroautophagy. Cell Res 24:24–41 24366339 10.1038/cr.2013.168 PMC3879710

[CR32] Feng Z, Zhang L, Wang S, Hong Q (2020) Circular RNA circDLGAP4 exerts neuroprotective effects via modulating miR-134-5p/CREB pathway in Parkinson’s disease. Biochem Biophys Res Commun 522:388–394 31761328 10.1016/j.bbrc.2019.11.102

[CR33] Fernández-de Frutos M, Galán-Chilet I, Goedeke L, Kim B, Pardo-Marqués V, Pérez-García A, Herrero JI, Fernández-Hernando C, Kim J, Ramírez CM (2019) MicroRNA 7 impairs insulin signaling and regulates Aβ levels through posttranscriptional regulation of the insulin receptor substrate 2, insulin receptor, insulin-degrading enzyme, and liver X receptor pathway. Mol Cell Biol 39:e00170-e1119 31501273 10.1128/MCB.00170-19 PMC6817752

[CR34] Fragkouli A, Doxakis E (2014) miR-7 and miR-153 protect neurons against MPP+-induced cell death via upregulation of mTOR pathway. Front Cell Neurosci 8:182 25071443 10.3389/fncel.2014.00182 PMC4080263

[CR35] Fujita N, Hayashi-Nishino M, Fukumoto H, Omori H, Yamamoto A, Noda T, Yoshimori T (2008) An Atg4B mutant hampers the lipidation of LC3 paralogues and causes defects in autophagosome closure. Mol Biol Cell 19:4651–4659 18768752 10.1091/mbc.E08-03-0312 PMC2575160

[CR36] Ge B, Wu H, Shao D, Li S, Li F (2019) Interfering with miR-24 alleviates rotenone-induced dopaminergic neuron injury via enhancing autophagy by upregulating DJ-1. Aging Pathobiol Ther 1:17–24

[CR37] Ge X, Guo M, Hu T, Li W, Huang S, Yin Z, Li Y, Chen F, Zhu L, Kang C (2020) Increased microglial exosomal miR-124-3p alleviates neurodegeneration and improves cognitive outcome after rmTBI. Mol Ther 28:503–522 31843449 10.1016/j.ymthe.2019.11.017 PMC7001001

[CR38] Gong X, Wang H, Ye Y, Shu Y, Deng Y, He X, Lu G, Zhang S (2016) miR-124 regulates cell apoptosis and autophagy in dopaminergic neurons and protects them by regulating AMPK/mTOR pathway in Parkinson’s disease. Am J Transl Res 8:2127 27347320 PMC4891425

[CR39] Gourvest M, Brousset P, Bousquet M (2019) Long noncoding RNAs in acute myeloid leukemia: functional characterization and clinical relevance. Cancers (basel). 10.1038/cdd.2015.172 31653018 10.3390/cancers11111638 PMC6896193

[CR40] Green D, Oguin TH, Martinez J (2016) The clearance of dying cells: table for two. Cell Death Differ 23:915–926 26990661 10.1038/cdd.2015.172 PMC4987729

[CR41] Gregory RI, Yan K-P, Amuthan G, Chendrimada T, Doratotaj B, Cooch N, Shiekhattar R (2004) The Microprocessor complex mediates the genesis of microRNAs. Nature 432:235–240 15531877 10.1038/nature03120

[CR42] Gu H, Li L, Cui C, Zhao Z, Song G (2017) Overexpression of let-7a increases neurotoxicity in a PC12 cell model of Alzheimer’s disease via regulating autophagy. Exp Ther Med 14:3688–3698 29042965 10.3892/etm.2017.4977 PMC5639351

[CR43] Guo JU, Agarwal V, Guo H, Bartel DP (2014) Expanded identification and characterization of mammalian circular RNAs. Genome Biol 15:1–14 10.1186/s13059-014-0409-z PMC4165365 25070500

[CR44] Guo C-C, Jiao C-H, Gao Z-M (2018a) Silencing of LncRNA BDNF-AS attenuates Aβ25-35-induced neurotoxicity in PC12 cells by suppressing cell apoptosis and oxidative stress. Neurol Res 40:795–804 29902125 10.1080/01616412.2018.1480921

[CR45] Guo F, Liu X, Cai H, Le W (2018b) Autophagy in neurodegenerative diseases: pathogenesis and therapy. Brain Pathol 28:3–13 28703923 10.1111/bpa.12545 PMC5739982

[CR46] Hamano T, Gendron TF, Causevic E, Yen SH, Lin WL, Isidoro C, DeTure M, Ko L-w (2008) Autophagic-lysosomal perturbation enhances tau aggregation in transfectants with induced wild-type tau expression. Eur J Neurosci 27:1119–1130 18294209 10.1111/j.1460-9568.2008.06084.x

[CR47] Han D, Dong X, Zheng D, Nao J (2020) MiR-124 and the underlying therapeutic promise of neurodegenerative disorders. Front Pharmacol 10:1555 32009959 10.3389/fphar.2019.01555 PMC6978711

[CR48] Hara T, Nakamura K, Matsui M, Yamamoto A, Nakahara Y, Suzuki-Migishima R, Yokoyama M, Mishima K, Saito I, Okano H (2006) Suppression of basal autophagy in neural cells causes neurodegenerative disease in mice. Nature 441:885–889 16625204 10.1038/nature04724

[CR49] He W, Chi S, Jin X, Lu J, Zheng W, Yan J, Zhang D (2020) Long non-coding RNA BACE1-AS modulates isoflurane-induced neurotoxicity to Alzheimer’s Disease through sponging miR-214-3p. Neurochem Res 45:2324–2335 32681443 10.1007/s11064-020-03091-2

[CR50] Hegarty SV, Sullivan AM, O’Keeffe GW (2018) Inhibition of miR-181a promotes midbrain neuronal growth through a Smad1/5-dependent mechanism: implications for Parkinson’s disease. Neuronal Signal. 10.1042/NS20170181 32714583 10.1042/NS20170181 PMC7371012

[CR51] Ho T-T, Zhou N, Huang J, Koirala P, Xu M, Fung R, Wu F, Mo Y-Y (2015) Targeting non-coding RNAs with the CRISPR/Cas9 system in human cell lines. Nucleic Acids Res 43:e17–e17 25414344 10.1093/nar/gku1198 PMC4330338

[CR52] Hu G, Shi Z, Shao W, Xu B (2022) MicroRNA-214–5p involves in the protection effect of dexmedetomidine against neurological injury in Alzheimer’s disease via targeting the suppressor of zest 12. Brain Res Bull 178:164–172 34715270 10.1016/j.brainresbull.2021.10.016

[CR53] Huang Z, Zhao J, Wang W, Zhou J, Zhang J (2020) Depletion of LncRNA NEAT1 rescues mitochondrial dysfunction through NEDD4L-dependent PINK1 degradation in animal models of Alzheimer’s disease. Front Cell Neurosci 14:28 32140098 10.3389/fncel.2020.00028 PMC7043103

[CR54] Itakura E, Kishi-Itakura C, Mizushima N (2012) The hairpin-type tail-anchored SNARE syntaxin 17 targets to autophagosomes for fusion with endosomes/lysosomes. Cell 151:1256–1269 23217709 10.1016/j.cell.2012.11.001

[CR55] Jiang Y, Xu N (2023) The emerging role of autophagy-associated lncRNAs in the pathogenesis of neurodegenerative diseases. Int J Mol Sci 24:9686 37298636 10.3390/ijms24119686 PMC10254068

[CR56] Jung CH, Jun CB, Ro S-H, Kim Y-M, Otto NM, Cao J, Kundu M, Kim D-H (2009) ULK-Atg13-FIP200 complexes mediate mTOR signaling to the autophagy machinery. Mol Biol Cell 20:1992–2003 19225151 10.1091/mbc.E08-12-1249 PMC2663920

[CR57] Kabeya Y, Mizushima N, Ueno T, Yamamoto A, Kirisako T, Noda T, Kominami E, Ohsumi Y, Yoshimori T (2000) LC3, a mammalian homologue of yeast Apg8p, is localized in autophagosome membranes after processing. EMBO J 19:5720–5728 11060023 10.1093/emboj/19.21.5720 PMC305793

[CR58] Ke S, Yang Z, Yang F, Wang X, Tan J, Liao B (2019) Long noncoding RNA NEAT1 aggravates Aβ-induced neuronal damage by targeting miR-107 in Alzheimer’s disease. Yonsei Med J 60:640–650 31250578 10.3349/ymj.2019.60.7.640 PMC6597469

[CR59] Kim J, Yoon H, Ramírez CM, Lee S-M, Hoe H-S, Fernández-Hernando C, Kim J (2012) MiR-106b impairs cholesterol efflux and increases Aβ levels by repressing ABCA1 expression. Exp Neurol 235:476–483 22119192 10.1016/j.expneurol.2011.11.010 PMC3328628

[CR60] Komatsu M, Waguri S, Chiba T, Murata S, Iwata J-I, Tanida I, Ueno T, Koike M, Uchiyama Y, Kominami E (2006) Loss of autophagy in the central nervous system causes neurodegeneration in mice. Nature 441:880–884 16625205 10.1038/nature04723

[CR61] Kraus TF, Haider M, Spanner J, Steinmaurer M, Dietinger V, Kretzschmar HA (2017) Altered long noncoding RNA expression precedes the course of Parkinson’s disease—a preliminary report. Mol Neurobiol 54:2869–2877 27021022 10.1007/s12035-016-9854-x

[CR62] Kuhn HG, Palmer TD, Fuchs E (2001) Adult neurogenesis: a compensatory mechanism for neuronal damage. Eur Arch Psychiatry Clin Neurosci 251:152–158 11697579 10.1007/s004060170035

[CR63] Lagos-Quintana M, Rauhut R, Yalcin A, Meyer J, Lendeckel W, Tuschl T (2002) Identification of tissue-specific microRNAs from mouse. Curr Biol 12:735–739 12007417 10.1016/s0960-9822(02)00809-6

[CR64] Lang Y, Li Y, Yu H, Lin L, Chen X, Wang S, Zhang H (2020) HOTAIR drives autophagy in midbrain dopaminergic neurons in the substantia nigra compacta in a mouse model of Parkinson’s disease by elevating NPTX2 via miR-221-3p binding. Aging (albany NY) 12:7660 32396526 10.18632/aging.103028 PMC7244061

[CR65] Levine B, Kroemer G (2008) Autophagy in the pathogenesis of disease. Cell 132:27–42 18191218 10.1016/j.cell.2007.12.018 PMC2696814

[CR66] Li S, Lv X, Zhai K, Xu R, Zhang Y, Zhao S, Qin X, Yin L, Lou J (2016) MicroRNA-7 inhibits neuronal apoptosis in a cellular Parkinson’s disease model by targeting Bax and Sirt2. Am J Transl Res 8:993 27158385 PMC4846942

[CR67] Li W, Jiang Y, Wang Y, Yang S, Bi X, Pan X, Ma A (2018) MiR-181b regulates autophagy in a model of Parkinson’s disease by targeting the PTEN/Akt/mTOR signaling pathway. Neurosci Lett 675:83–88 29608948 10.1016/j.neulet.2018.03.041

[CR68] Li Q, Wang Y, Peng W, Jia Y, Tang J, Li W, Zhang JH, Yang J (2019) MicroRNA-101a regulates autophagy phenomenon via the MAPK pathway to modulate Alzheimer’s-associated pathogenesis. Cell Transplant 28:1076–1084 31204500 10.1177/0963689719857085 PMC6728707

[CR69] Li Y, Fang J, Zhou Z, Zhou Q, Sun S, Jin Z, Xi Z, Wei J (2020) Downregulation of lncRNA BACE1-AS improves dopamine-dependent oxidative stress in rats with Parkinson’s disease by upregulating microRNA-34b-5p and downregulating BACE1. Cell Cycle 19:1158–1171 32308102 10.1080/15384101.2020.1749447 PMC7217373

[CR70] Liu Y, Song Y, Zhu X (2017) MicroRNA-181a regulates apoptosis and autophagy process in Parkinson’s disease by inhibiting p38 mitogen-activated protein kinase (MAPK)/c-Jun N-terminal kinases (JNK) signaling pathways. Med Sci Monit: Int Med J Exp Clin Res 23:1597 10.12659/MSM.900218 PMC5386441 28365714

[CR71] Liu W, Li L, Liu S, Wang Z, Kuang H, Xia Y, Tang C, Yin D (2019) MicroRNA expression profiling screen miR-3557/324-targeted CaMK/mTOR in the rat striatum of Parkinson’s disease in regular aerobic exercise. BioMed Res Int. 10.1155/2019/7654798 31309116 10.1155/2019/7654798 PMC6594308

[CR72] Liu J, Liu D, Zhao B, Jia C, Lv Y, Liao J, Li K (2020) Long non-coding RNA NEAT1 mediates MPTP/MPP+-induced apoptosis via regulating the miR-124/KLF4 axis in Parkinson’s disease. Open Life Sci 15:665–676 33817255 10.1515/biol-2020-0069 PMC7747504

[CR73] Long JM, Lahiri DK (2011) MicroRNA-101 downregulates Alzheimer’s amyloid-β precursor protein levels in human cell cultures and is differentially expressed. Biochem Biophys Res Commun 404:889–895 21172309 10.1016/j.bbrc.2010.12.053 PMC3372402

[CR74] Lu W, Lin J, Zheng D, Hong C, Ke L, Wu X, Chen P (2020) Overexpression of MicroRNA-133a inhibits apoptosis and autophagy in a cell model of Parkinson’s disease by downregulating ras-related C3 botulinum toxin substrate 1 (RAC1). Med Sci Monitor: Int Med J Exp Clin Res 26:e922032–e922031 10.12659/MSM.922032 PMC7409387 32713934

[CR75] Lucin KM, O’Brien CE, Bieri G, Czirr E, Mosher KI, Abbey RJ, Mastroeni DF, Rogers J, Spencer B, Masliah E (2013) Microglial beclin 1 regulates retromer trafficking and phagocytosis and is impaired in Alzheimer’s disease. Neuron 79:873–886 24012002 10.1016/j.neuron.2013.06.046 PMC3779465

[CR76] Lv Q, Zhong Z, Hu B, Yan S, Yan Y, Zhang J, Shi T, Jiang L, Li W, Huang W (2021) MicroRNA-3473b regulates the expression of TREM2/ULK1 and inhibits autophagy in inflammatory pathogenesis of Parkinson’s disease. J Neurochem 157:599–610 33448372 10.1111/jnc.15299

[CR77] Manzoni C, Mamais A, Dihanich S, Abeti R, Soutar MP, Plun-Favreau H, Giunti P, Tooze SA, Bandopadhyay R, Lewis PA (2013) Inhibition of LRRK2 kinase activity stimulates macroautophagy. Biochim Biophys Acta (BBA) Mol Cell Res 1833:2900–2910 10.1016/j.bbamcr.2013.07.020 PMC3898616 23916833

[CR78] Marquez RT, Xu L (2012) Bcl-2: Beclin 1 complex: multiple, mechanisms regulating autophagy/apoptosis toggle switch. Am J Cancer Res 2:214 22485198 PMC3304572

[CR79] Martyniszyn L, Szulc L, Boratyńska A, Niemiałtowski MG (2011) Beclin 1 is involved in regulation of apoptosis and autophagy during replication of ectromelia virus in permissive L929 cells. Arch Immunol Ther Exp 59:463–471 10.1007/s00005-011-0149-7 21972018

[CR80] Menzies FM, Fleming A, Rubinsztein DC (2015) Compromised autophagy and neurodegenerative diseases. Nat Rev Neurosci 16:345–357 25991442 10.1038/nrn3961

[CR81] Mishima T, Mizuguchi Y, Kawahigashi Y, Takizawa T, Takizawa T (2007) RT-PCR-based analysis of microRNA (miR-1 and-124) expression in mouse CNS. Brain Res 1131:37–43 17182009 10.1016/j.brainres.2006.11.035

[CR82] Nah J, Yuan J, Jung Y-K (2015) Autophagy in neurodegenerative diseases: from mechanism to therapeutic approach. Mol Cells 38:381 25896254 10.14348/molcells.2015.0034 PMC4443278

[CR83] Narendra D, Tanaka A, Suen D-F, Youle RJ (2008) Parkin is recruited selectively to impaired mitochondria and promotes their autophagy. J Cell Biol 183:795–803 19029340 10.1083/jcb.200809125 PMC2592826

[CR84] Nikoletopoulou V, Markaki M, Palikaras K, Tavernarakis N (2013) Crosstalk between apoptosis, necrosis and autophagy. Biochim Biophys Acta (BBA) Mol Cell Res 1833:3448–3459 10.1016/j.bbamcr.2013.06.001 23770045

[CR85] Nojima T, Proudfoot NJ (2022) Mechanisms of lncRNA biogenesis as revealed by nascent transcriptomics. Nat Rev Mol Cell Biol 23:389–406 35079163 10.1038/s41580-021-00447-6

[CR86] Obara K, Ohsumi Y (2008) Dynamics and function of PtdIns (3) P in autophagy. Autophagy 4:952–954 18769109 10.4161/auto.6790

[CR87] Ohsumi Y, Mizushima N (2004) Two ubiquitin-like conjugation systems essential for autophagy. Sem Cell Dev Biol 15:231–236 10.1016/j.semcdb.2003.12.004 15209383

[CR88] Omura T, Nomura L, Watanabe R, Nishiguchi H, Yamamoto K, Imai S, Nakagawa S, Itohara K, Yonezawa A, Nakagawa T (2021) MicroRNA-101 regulates 6-hydroxydopamine-induced cell death by targeting suppressor/enhancer lin-12-like in SH-SY5Y cells. Front Mol Neurosci. 10.3389/fnmol.2021.748026 34955743 10.3389/fnmol.2021.748026 PMC8695805

[CR89] Patergnani S, Pinton P (2015) Mitophagy and mitochondrial balance. Mitochondrial Regul. 10.1007/978-1-4939-1875-1_15 10.1007/978-1-4939-1875-1_15 25308497

[CR90] Patergnani S, Guzzo S, Mangolini A, dell’Atti L, Pinton P, Aguiari G (2020) The induction of AMPK-dependent autophagy leads to P53 degradation and affects cell growth and migration in kidney cancer cells. Exp Cell Res 395:112190 32717219 10.1016/j.yexcr.2020.112190

[CR91] Pattingre S, Tassa A, Qu X, Garuti R, Liang XH, Mizushima N, Packer M, Schneider MD, Levine B (2005) Bcl-2 antiapoptotic proteins inhibit Beclin 1-dependent autophagy. Cell 122:927–939 16179260 10.1016/j.cell.2005.07.002

[CR92] Plaza-Zabala A, Sierra-Torre V, Sierra A (2017) Autophagy and microglia: novel partners in neurodegeneration and aging. Int J Mol Sci 18:598 28282924 10.3390/ijms18030598 PMC5372614

[CR93] Puri C, Renna M, Bento CF, Moreau K, Rubinsztein DC (2013) Diverse autophagosome membrane sources coalesce in recycling endosomes. Cell 154:1285–1299 24034251 10.1016/j.cell.2013.08.044 PMC3791395

[CR94] Qian C, Ye Y, Mao H, Yao L, Sun X, Wang B, Zhang H, Xie L, Zhang H, Zhang Y (2019) Downregulated lncRNA-SNHG1 enhances autophagy and prevents cell death through the miR-221/222/p27/mTOR pathway in Parkinson’s disease. Exp Cell Res 384:111614 31499060 10.1016/j.yexcr.2019.111614

[CR95] Qin X, Zhang X, Li P, Wang M, Yan L, Pan P, Zhang H, Hong X, Liu M, Bao Z (2021a) MicroRNA-185 activates PI3K/AKT signalling pathway to alleviate dopaminergic neuron damage via targeting IGF1 in Parkinson’s disease. J Drug Target 29:875–883 33560148 10.1080/1061186X.2021.1886300

[CR96] Qin Y, Qiu J, Wang P, Liu J, Zhao Y, Jiang F, Lou H (2021b) Impaired autophagy in microglia aggravates dopaminergic neurodegeneration by regulating NLRP3 inflammasome activation in experimental models of Parkinson’s disease. Brain Behav Immun 91:324–338 33039664 10.1016/j.bbi.2020.10.010

[CR97] Querfurth HW, LaFerla FM (2010) Alzheimer’s disease. N Engl J Med 362:329–344 20107219 10.1056/NEJMra0909142

[CR98] Rahimmi A, Peluso I, Rajabi A, Hassanzadeh K (2019) miR-185 and SEPT5 genes may contribute to Parkinson’s disease pathophysiology. Oxid Med Cell Longev. 10.1155/2019/5019815 31814881 10.1155/2019/5019815 PMC6878792

[CR99] Ravikumar B, Duden R, Rubinsztein DC (2002) Aggregate-prone proteins with polyglutamine and polyalanine expansions are degraded by autophagy. Hum Mol Genet 11:1107–1117 11978769 10.1093/hmg/11.9.1107

[CR100] Ravikumar B, Acevedo-Arozena A, Imarisio S, Berger Z, Vacher C, O’Kane CJ, Brown SD, Rubinsztein DC (2005) Dynein mutations impair autophagic clearance of aggregate-prone proteins. Nat Genet 37:771–776 15980862 10.1038/ng1591

[CR101] Riebisch AK, Mühlen S, Beer YY, Schmitz I (2021) Autophagy—a story of bacteria interfering with the host cell degradation machinery. Pathogens 10:110 33499114 10.3390/pathogens10020110 PMC7911818

[CR102] Rodríguez-Martín T, Cuchillo-Ibáñez I, Noble W, Nyenya F, Anderton BH, Hanger DP (2013) Tau phosphorylation affects its axonal transport and degradation. Neurobiol Aging 34:2146–2157 23601672 10.1016/j.neurobiolaging.2013.03.015 PMC3684773

[CR103] Rodriguez-Ortiz CJ, Prieto GA, Martini AC, Forner S, Trujillo-Estrada L, LaFerla FM, Baglietto-Vargas D, Cotman CW, Kitazawa M (2020) miR-181a negatively modulates synaptic plasticity in hippocampal cultures and its inhibition rescues memory deficits in a mouse model of Alzheimer’s disease. Aging Cell 19:e13118 32087004 10.1111/acel.13118 PMC7059142

[CR104] Sala G, Stefanoni G, Arosio A, Riva C, Melchionda L, Saracchi E, Fermi S, Brighina L, Ferrarese C (2014) Reduced expression of the chaperone-mediated autophagy carrier hsc70 protein in lymphomonocytes of patients with Parkinson’s disease. Brain Res 1546:46–52 24361989 10.1016/j.brainres.2013.12.017

[CR105] Sang Q, Liu X, Wang L, Qi L, Sun W, Wang W, Sun Y, Zhang H (2018) CircSNCA downregulation by pramipexole treatment mediates cell apoptosis and autophagy in Parkinson’s disease by targeting miR-7. Aging (albany NY) 10:1281 29953413 10.18632/aging.101466 PMC6046232

[CR106] Scarffe LA, Stevens DA, Dawson VL, Dawson TM (2014) Parkin and PINK1: much more than mitophagy. Trends Neurosci 37:315–324 24735649 10.1016/j.tins.2014.03.004 PMC4075431

[CR107] Shah SZA, Zhao D, Hussain T, Sabir N, Yang L (2018) Regulation of microRNAs-mediated autophagic flux: a new regulatory avenue for neurodegenerative diseases with focus on prion diseases. Front Aging Neurosci 10:139 29867448 10.3389/fnagi.2018.00139 PMC5962651

[CR108] Shao Y, Gao Z, Feldman T, Jiang X (2007) Stimulation of ATG12-ATG5 conjugation by ribonucleic acid. Autophagy 3:10–16 16963840 10.4161/auto.3270

[CR109] Shibutani ST, Saitoh T, Nowag H, Münz C, Yoshimori T (2015) Autophagy and autophagy-related proteins in the immune system. Nat Immunol 16:1014–1024 26382870 10.1038/ni.3273

[CR110] Sierra A, Abiega O, Shahraz A, Neumann H (2013) Janus-faced microglia: beneficial and detrimental consequences of microglial phagocytosis. Front Cell Neurosci 7:6 23386811 10.3389/fncel.2013.00006 PMC3558702

[CR111] Smith PY, Hernandez-Rapp J, Jolivette F, Lecours C, Bisht K, Goupil C, Dorval V, Parsi S, Morin F, Planel E (2015) miR-132/212 deficiency impairs tau metabolism and promotes pathological aggregation in vivo. Hum Mol Genet 24:6721–6735 26362250 10.1093/hmg/ddv377 PMC4634376

[CR112] Song C, Zhang Y, Huang W, Shi J, Huang Q, Jiang M, Qiu Y, Wang T, Chen H, Wang H (2022) Circular RNA Cwc27 contributes to Alzheimer’s disease pathogenesis by repressing Pur-α activity. Cell Death Differ 29:393–406 34504314 10.1038/s41418-021-00865-1 PMC8817017

[CR113] Spencer B, Potkar R, Trejo M, Rockenstein E, Patrick C, Gindi R, Adame A, Wyss-Coray T, Masliah E (2009) Beclin 1 gene transfer activates autophagy and ameliorates the neurodegenerative pathology in α-synuclein models of Parkinson’s and Lewy body diseases. J Neurosci 29:13578–13588 19864570 10.1523/JNEUROSCI.4390-09.2009 PMC2812014

[CR114] Stein CS, McLendon JM, Witmer NH, Boudreau RL (2022) Modulation of miR-181 influences dopaminergic neuronal degeneration in a mouse model of Parkinson’s disease. Mol Ther Nucleic Acids 28:1–15 35280925 10.1016/j.omtn.2022.02.007 PMC8899134

[CR115] Su P, Zhang J, Wang D, Zhao F, Cao Z, Aschner M, Luo W (2016) The role of autophagy in modulation of neuroinflammation in microglia. Neuroscience 319:155–167 26827945 10.1016/j.neuroscience.2016.01.035

[CR116] Subramanian M, Hyeon SJ, Das T, Suh YS, Kim YK, Lee J-S, Song EJ, Ryu H, Yu K (2021) UBE4B, a microRNA-9 target gene, promotes autophagy-mediated Tau degradation. Nat Commun 12:1–15 34078905 10.1038/s41467-021-23597-9 PMC8172564

[CR117] Sun S, Han X, Li X, Song Q, Lu M, Jia M, Ding J, Hu G (2018) MicroRNA-212-5p prevents dopaminergic neuron death by inhibiting SIRT2 in MPTP-induced mouse model of Parkinson’s disease. Front Mol Neurosci 11:381 30364275 10.3389/fnmol.2018.00381 PMC6193094

[CR118] Surendran S, Rajasankar S (2010) Parkinson’s disease: oxidative stress and therapeutic approaches. Neurol Sci 31:531–540 20221655 10.1007/s10072-010-0245-1

[CR119] Suzuki H, Tsukahara T (2014) A view of pre-mRNA splicing from RNase R resistant RNAs. Int J Mol Sci 15:9331–9342 24865493 10.3390/ijms15069331 PMC4100097

[CR120] Talepoor Ardakani M, Rostamian Delavar M, Baghi M, Nasr-Esfahani MH, Kiani-Esfahani A, Ghaedi K (2019) Upregulation of miR-200a and miR-204 in MPP+-treated differentiated PC12 cells as a model of Parkinson’s disease. Mol Genet Genomic Med 7:e548 30712312 10.1002/mgg3.548 PMC6418372

[CR121] Tan C-C, Yu J-T, Tan M-S, Jiang T, Zhu X-C, Tan L (2014) Autophagy in aging and neurodegenerative diseases: implications for pathogenesis and therapy. Neurobiol Aging 35:941–957 24360503 10.1016/j.neurobiolaging.2013.11.019

[CR122] Tang Z-B, Chen H-P, Zhong D, Song J-H, Cao J-W, Zhao M-Q, Han B-C, Duan Q, Sheng X-M, Yao J-L (2022) LncRNA RMRP accelerates autophagy-mediated neurons apoptosis through miR-3142/TRIB3 signaling axis in alzheimer’s disease. Brain Res 1785:147884 35304105 10.1016/j.brainres.2022.147884

[CR123] Titze-de-Almeida R, Titze-de-Almeida SS (2018) miR-7 replacement therapy in Parkinson’s disease. Curr Gene Ther 18:143–153 29714132 10.2174/1566523218666180430121323

[CR124] Ułamek-Kozioł M, Furmaga-Jabłońska W, Januszewski S, Brzozowska J, Ściślewska M, Jabłoński M, Pluta R (2013) Neuronal autophagy: self-eating or self-cannibalism in Alzheimer’s disease. Neurochem Res 38:1769–1773 23737325 10.1007/s11064-013-1082-4 PMC3732752

[CR125] Valera E, Spencer B, Mott J, Trejo M, Adame A, Mante M, Rockenstein E, Troncoso JC, Beach TG, Masliah E (2017) MicroRNA-101 modulates autophagy and oligodendroglial alpha-synuclein accumulation in multiple system atrophy. Front Mol Neurosci 10:329 29089869 10.3389/fnmol.2017.00329 PMC5650998

[CR126] Wang H, Liu J, Zong Y, Xu Y, Deng W, Zhu H, Liu Y, Ma C, Huang L, Zhang L (2010) miR-106b aberrantly expressed in a double transgenic mouse model for Alzheimer’s disease targets TGF-β type II receptor. Brain Res 1357:166–174 20709030 10.1016/j.brainres.2010.08.023

[CR127] Wang J-Z, Xia Y-Y, Grundke-Iqbal I, Iqbal K (2013) Abnormal hyperphosphorylation of tau: sites, regulation, and molecular mechanism of neurofibrillary degeneration. J Alzheimer’s Dis 33:S123–S139 22710920 10.3233/JAD-2012-129031

[CR128] Wang H, Ye Y, Zhu Z, Mo L, Lin C, Wang Q, Wang H, Gong X, He X, Lu G (2016) MiR-124 regulates apoptosis and autophagy process in MPTP model of Parkinson’s disease by targeting to B im. Brain Pathol 26:167–176 25976060 10.1111/bpa.12267 PMC8029438

[CR129] Wang S, Zhang X, Guo Y, Rong H, Liu T (2017a) The long noncoding RNA HOTAIR promotes Parkinson’s disease by upregulating LRRK2 expression. Oncotarget 8:24449 28445933 10.18632/oncotarget.15511 PMC5421861

[CR130] Wang Y, Veremeyko T, Wong AH-K, El Fatimy R, Wei Z, Cai W, Krichevsky AM (2017b) Downregulation of miR-132/212 impairs S-nitrosylation balance and induces tau phosphorylation in Alzheimer’s disease. Neurobiol Aging 51:156–166 28089352 10.1016/j.neurobiolaging.2016.12.015

[CR131] Wang Z, Sun L, Jia K, Wang H, Wang X (2019) miR-9-5p modulates the progression of Parkinson’s disease by targeting SIRT1. Neurosci Lett 701:226–233 30826419 10.1016/j.neulet.2019.02.038

[CR132] Wang R, Yao J, Gong F, Chen S, He Y, Hu C, Li C (2021a) miR-29c-3p regulates TET2 expression and inhibits autophagy process in Parkinson’s disease models. Genes Cells 26:684–697 34086379 10.1111/gtc.12877

[CR133] Wang W, Lv R, Zhang J, Liu Y (2021b) circSAMD4A participates in the apoptosis and autophagy of dopaminergic neurons via the miR-29c-3p-mediated AMPK/mTOR pathway in Parkinson’s disease. Mol Med Rep 24:1–10 10.3892/mmr.2021.12179 PMC8170871 34080649

[CR134] Wang Z-Y, Wen Z-J, Xu H-M, Zhang Y, Zhang Y-F (2022) Exosomal noncoding RNAs in central nervous system diseases: biological functions and potential clinical applications. Front Mol Neurosci 15:1004221 36438184 10.3389/fnmol.2022.1004221 PMC9681831

[CR135] Wen Z, Zhang J, Tang P, Tu N, Wang K, Wu G (2018) Overexpression of miR-185 inhibits autophagy and apoptosis of dopaminergic neurons by regulating the AMPK/mTOR signaling pathway in Parkinson’s disease. Mol Med Rep 17:131–137 29115479 10.3892/mmr.2017.7897 PMC5780076

[CR136] Xiao X, Tan Z, Jia M, Zhou X, Wu K, Ding Y, Li W (2021) Long noncoding RNA SNHG1 knockdown ameliorates apoptosis, oxidative stress and inflammation in models of Parkinson’s disease by inhibiting the miR-125b-5p/MAPK1 Axis. Neuropsychiatr Dis Treat 17:1153 33911864 10.2147/NDT.S286778 PMC8075359

[CR137] Xie S-P, Zhou F, Li J, Duan S-J (2019) NEAT1 regulates MPP+-induced neuronal injury by targeting miR-124 in neuroblastoma cells. Neurosci Lett 708:134340 31228597 10.1016/j.neulet.2019.134340

[CR138] Xu X, Cui L, Zhong W, Cai Y (2020) Autophagy-associated lncRNAs: promising targets for neurological disease diagnosis and therapy. Neural Plast. 10.1155/2020/8881687 33029125 10.1155/2020/8881687 PMC7528122

[CR139] Xue J, Patergnani S, Giorgi C, Suarez J, Goto K, Bononi A, Tanji M, Novelli F, Pastorino S, Xu R (2020) Asbestos induces mesothelial cell transformation via HMGB1-driven autophagy. Proc Natl Acad Sci 117:25543–25552 32999071 10.1073/pnas.2007622117 PMC7568322

[CR140] Yamamoto A, Yue Z (2014) Autophagy and its normal and pathogenic states in the brain. Annu Rev Neurosci 37:55–78 24821313 10.1146/annurev-neuro-071013-014149

[CR141] Yan W, Chen Z-Y, Chen J-Q, Chen H-M (2018) LncRNA NEAT1 promotes autophagy in MPTP-induced Parkinson’s disease through stabilizing PINK1 protein. Biochem Biophys Res Commun 496:1019–1024 29287722 10.1016/j.bbrc.2017.12.149

[CR142] Yao L, Zhu Z, Wu J, Zhang Y, Zhang H, Sun X, Qian C, Wang B, Xie L, Zhang S (2019) MicroRNA-124 regulates the expression of p62/p38 and promotes autophagy in the inflammatory pathogenesis of Parkinson’s disease. FASEB J 33:8648–8665 30995872 10.1096/fj.201900363R

[CR143] Yu Y, Zhang Y (2013) Desflurane accelerates neuronal cytotoxicity of Aβ by downregulating miR-214. Neurosci Lett 554:28–33 24021802 10.1016/j.neulet.2013.08.063

[CR144] Yu WH, Cuervo AM, Kumar A, Peterhoff CM, Schmidt SD, Lee J-H, Mohan PS, Mercken M, Farmery MR, Tjernberg LO (2005) Macroautophagy—a novel β-amyloid peptide-generating pathway activated in Alzheimer’s disease. J Cell Biol 171:87–98 16203860 10.1083/jcb.200505082 PMC2171227

[CR145] Zhang Y, Li Q, Liu C, Gao S, Ping H, Wang J, Wang P (2016a) MiR-214-3p attenuates cognition defects via the inhibition of autophagy in SAMP8 mouse model of sporadic Alzheimer’s disease. Neurotoxicology 56:139–149 27397902 10.1016/j.neuro.2016.07.004

[CR146] Zhang Y, Liu C, Wang J, Li Q, Ping H, Gao S, Wang P (2016b) MiR-299-5p regulates apoptosis through autophagy in neurons and ameliorates cognitive capacity in APPswe/PS1dE9 mice. Sci Rep 6:1–14 27080144 10.1038/srep24566 PMC4832239

[CR147] Zhang X, Hong R, Chen W, Xu M, Wang L (2019) The role of long noncoding RNA in major human disease. Bioorg Chem 92:103214 31499258 10.1016/j.bioorg.2019.103214

[CR148] Zhang L, Wang J, Liu Q, Xiao Z, Dai Q (2020a) Knockdown of long non-coding RNA AL049437 mitigates MPP+-induced neuronal injury in SH-SY5Y cells via the microRNA-205-5p/MAPK1 axis. Neurotoxicology 78:29–35 32057949 10.1016/j.neuro.2020.02.004

[CR149] Zhang Y, Xia Q, Lin J (2020b) LncRNA H19 attenuates apoptosis in MPTP-induced Parkinson’s disease through regulating miR-585-3p/PIK3R3. Neurochem Res 45:1700–1710 32356199 10.1007/s11064-020-03035-w

[CR150] Zhang L, Fang Y, Zhao X, Zheng Y, Ma Y, Li S, Huang Z, Li L (2021a) miR-204 silencing reduces mitochondrial autophagy and ROS production in a murine AD model via the TRPML1-activated STAT3 pathway. Mol Therapy Nucleic Acids 24:822–831 10.1016/j.omtn.2021.02.010 PMC8121631 34026326

[CR151] Zhang M, He P, Bian Z (2021b) Long noncoding RNAs in neurodegenerative diseases: pathogenesis and potential implications as clinical biomarkers. Front Mol Neurosci 14:685143 34421536 10.3389/fnmol.2021.685143 PMC8371338

[CR152] Zhang Q, Huang X-M, Liao J-X, Dong Y-K, Zhu J-L, He C-C, Huang J, Tang Y-W, Wu D, Tian J-Y (2021c) LncRNA HOTAIR promotes neuronal damage through facilitating NLRP3 mediated-pyroptosis activation in Parkinson’s disease via regulation of miR-326/ELAVL1 axis. Cell Mol Neurobiol 41:1773–1786 32968928 10.1007/s10571-020-00946-8 PMC11444004

[CR153] Zhang H, Liang J, Chen N (2022) The potential role of miRNA-regulated autophagy in Alzheimer’s disease. Int J Mol Sci 23:7789 35887134 10.3390/ijms23147789 PMC9317523

[CR154] Zhao XH, Wang YB, Yang J, Liu HQ, Wang LL (2019) MicroRNA-326 suppresses iNOS expression and promotes autophagy of dopaminergic neurons through the JNK signaling by targeting XBP1 in a mouse model of Parkinson’s disease. J Cell Biochem 120:14995–15006 31135066 10.1002/jcb.28761

[CR155] Zhao J, Geng L, Chen Y, Wu C (2020a) SNHG1 promotes MPP+-induced cytotoxicity by regulating PTEN/AKT/mTOR signaling pathway in SH-SY5Y cells via sponging miR-153-3p. Biol Res 53:1–11 31907031 10.1186/s40659-019-0267-y PMC6943908

[CR156] Zhao J, Li H, Chang N (2020b) LncRNA HOTAIR promotes MPP+-induced neuronal injury in Parkinson’s disease by regulating the miR-874-5p/ATG10 axis. EXCLI J 19:1141 33013268 10.17179/excli2020-2286 PMC7527508

[CR157] Zhao J, Yang M, Li Q, Pei X, Zhu X (2020c) miR-132-5p regulates apoptosis and autophagy in MPTP model of Parkinson’s disease by targeting ULK1. NeuroReport 31:959–965 32658123 10.1097/WNR.0000000000001494

[CR158] Zhou B, Li L, Qiu X, Wu J, Xu L, Shao W (2020a) Long non-coding RNA ANRIL knockdown suppresses apoptosis and pro-inflammatory cytokines while enhancing neurite outgrowth via binding microRNA-125a in a cellular model of Alzheimer’s disease. Mol Med Rep 22:1489–1497 32626959 10.3892/mmr.2020.11203 PMC7339647

[CR159] Zhou J, Zhao Y, Li Z, Zhu M, Wang Z, Li Y, Xu T, Feng D, Zhang S, Tang F (2020b) miR-103a-3p regulates mitophagy in Parkinson’s disease through Parkin/Ambra1 signaling. Pharmacol Res 160:105197 32942015 10.1016/j.phrs.2020.105197

[CR160] Zhou Y, Ge Y, Liu Q, Li Y-X, Chao X, Guan J-J, Diwu Y-C, Zhang Q (2021) LncRNA BACE1-AS promotes autophagy-mediated neuronal damage through the miR-214-3p/ATG5 signalling axis in Alzheimer’s disease. Neuroscience 455:52–64 33197504 10.1016/j.neuroscience.2020.10.028

[CR161] Zhu W, Zhang H, Gao J, Xu Y (2021) Silencing of miR-497-5p inhibits cell apoptosis and promotes autophagy in Parkinson’s disease by upregulation of FGF2. Environ Toxicol 36:2302–2312 34459097 10.1002/tox.23344

